# Supplementary material for: A Global Screen for Assembly State Changes of the Mitotic Proteome by SEC-SWATH-MS
Source: Cell Syst. 2020 Feb 26;10(2):133–155.e6. doi: 10.1016/j.cels.2020.01.001 (PMC7042714; doi:10.1016/j.cels.2020.01.001)

Q9Y6M0 | TEST_HUMAN | PRSS21 ESP1 TEST1 UNQ266/PRO303
Monomer MW [kDa]: 34.884 Monomer expected elution fraction: 49

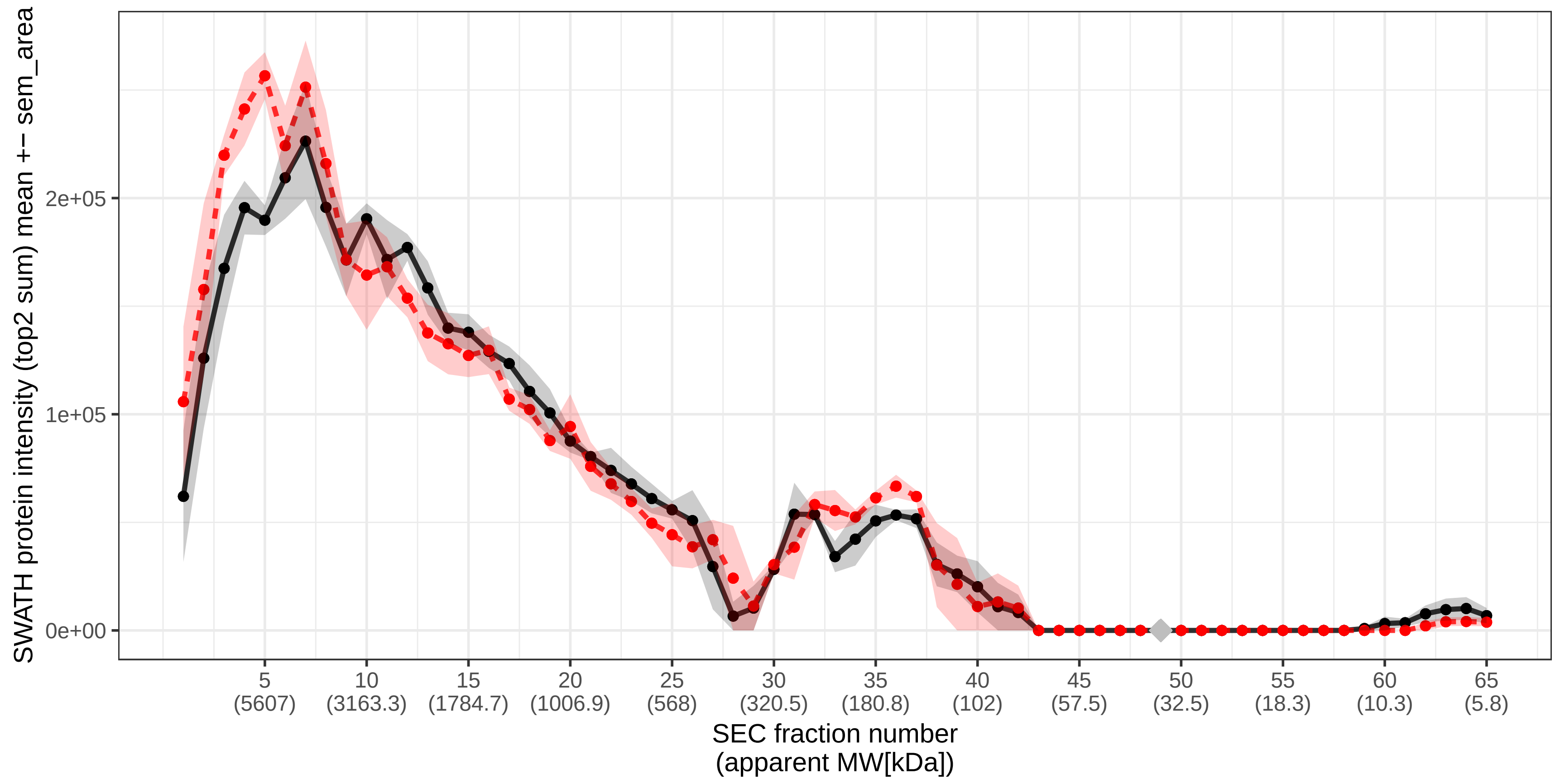

Supplement: Data S1. SEC-SWATH-MS Protein Chromatograms, Related to Figure 1 [file mmc6.zip › SECchrom_Q9Y6M0_TEST_HUMAN_PRSS21_ESP1_TEST1_UNQ266_PRO303.pdf]
